# Acute and adaptive cardiovascular and metabolic effects of passive heat therapy or high‐intensity interval training in patients with severe lower‐limb osteoarthritis

**DOI:** 10.14814/phy2.15699

**Published:** 2023-06-10

**Authors:** Brendon H. Roxburgh, Holly A. Campbell, James D. Cotter, Ulla Reymann, Michael J. A. Williams, David Gwynne‐Jones, Kate N. Thomas

**Affiliations:** ^1^ Department of Surgical Sciences Dunedin School of Medicine University of Otago Dunedin New Zealand; ^2^ School of Physical Education Sport and Exercise Sciences University of Otago Dunedin New Zealand; ^3^ HeartOtago University of Otago Dunedin New Zealand; ^4^ Department of Medicine Dunedin School of Medicine University of Otago Dunedin New Zealand; ^5^ Department of Orthopaedic Surgery Dunedin Hospital Southern District Health Board Dunedin New Zealand

**Keywords:** blood pressure, glycemic control, high‐intensity interval training, hot‐water immersion, osteoarthritis, passive heat therapy

## Abstract

Exercise is painful and difficult to perform for patients with severe lower‐limb osteoarthritis; consequently, reduced physical activity contributes to increased cardiometabolic disease risk. The aim of this study was to characterize the acute and adaptive cardiovascular and metabolic effects of two low or no impact therapies in patients with severe lower‐limb osteoarthritis: passive heat therapy (Heat) and high‐intensity interval training (HIIT) utilizing primarily the unaffected limbs, compared to a control intervention of home‐based exercise (Home). Participants completed up to 12 weeks of either Heat (20–30 min immersed in 40°C water followed by ~15‐min light resistance exercise), HIIT (6–8 × 60‐s intervals on a cross‐trainer or arm ergometer at ~90–100% peak $\dot{V}$O_2_) or Home (~15‐min light resistance exercise); all 3 sessions/week. Reductions in systolic (12 & 10 mm Hg), diastolic (7 & 4 mm Hg), and mean arterial (8 & 6 mm Hg) blood pressure (BP) were observed following one bout of Heat or HIIT exposure, lasting for the duration of the 20‐min monitoring period. Across the interventions (i.e., 12 weeks), resting systolic BP and diastolic BP decreased with Heat (−9 & ‐4 mm Hg; *p* < 0.001) and HIIT (−7 & ‐3 mm Hg; *p* ≤ 0.011), but not Home (0 & 0 mm Hg; *p* ≥ 0.785). The systolic and diastolic BP responses to an acute exposure of Heat or HIIT in the first intervention session were moderately correlated with adaptive responses across the intervention (*r* ≥ 0.54, *p* ≤ 0.005). Neither intervention improved indices of glycemic control (*p* = 0.310). In summary, both Heat and HIIT induced potent immediate and adaptive hypotensive effects, and the acute response was moderately predictive of the long‐term response.

## 1 INTRODUCTION

Osteoarthritis is the most common form of arthritis, with the lifetime risk of developing symptomatic osteoarthritis of the knee estimated at 45% (Murphy et al., 2008). Approximately 50% of patients with osteoarthritis are physically inactive, frequently citing joint pain as the main barrier (Kanavaki et al., 2017). Physical inactivity is a major global health concern due to its negative impact on risk factors for cardiovascular disease (CVD), such as hypertension, poor glycemic control, dyslipidemia, and obesity (Cecchini et al., 2010; Katzmarzyk et al., 2003; Lee et al., 2012). People suffering from osteoarthritis have a higher prevalence of these risk factors (Louati et al., 2015; Puenpatom & Victor, 2009), and therefore elevated rates of CVD (Wang et al., 2016), secondary to physical inactivity (Rahman et al., 2013). In particular, the prevalence of comorbid hypertension is estimated at 65% in patients with osteoarthritis (Marksabcdef & Allegranteeg, 2002). In addition, many of the pharmaceuticals used to treat osteoarthritis further increase blood pressure (BP) (Aljadhey et al., 2012; White, 2009). Hypertension significantly increases the risk of developing heart and kidney disease (McLean et al., 2013; World Health Organization, 2013), with high systolic BP also the leading risk factor for stroke (GBD 2019 Stroke Collaborators, 2021). Encouragingly, a meta‐analysis of 147 randomized controlled trials demonstrated that a 10 mm Hg reduction in systolic BP is associated with almost half the risk of stroke (Gaciong et al., 2013). Exercise is a critical component of first‐line therapy for managing hypertension (Gibbs et al., 2021); however, for patients with lower‐limb osteoarthritis, the weight‐bearing nature of traditional aerobic exercise (e.g., walking, cycling) is painful and sometimes impossible. Innovative, accessible, non‐pharmaceutical approaches to manage cardiovascular risk are evidently required.

Using the healthy upper limbs to perform exercise is one pragmatic approach for those encumbered with painful lower‐limb osteoarthritis. Indeed, the acute and adaptive cardiovascular and metabolic effects of arm exercise are similar to that of exercise using the lower limbs (Francois et al., 2017; Graham et al., 2016; Walker et al., 2000; Westhoff et al., 2008; Zwierska et al., 2005). Arm ergometry and elliptical training are both feasible options; elliptical trainers have similar biomechanical demands as walking (Burnfield et al., 2010) but with a more fluid, non‐impactful motion, plus the upper extremities are engaged to augment movement as much as preferred. Arm ergometry is also an alternative modality not requiring the use of lower limbs at all, allowing participants to perform aerobically‐demanding exercise, potentially pain free (Roxburgh et al., 2021). High‐intensity interval training (HIIT) has emerged as a safe and time‐efficient form of exercise for improving cardiovascular and metabolic health (Gibala et al., 2012; Weston et al., 2016); importantly, previously inactive adults *prefer* HIIT, compared to traditional continuous moderate‐intensity exercise training (Jung et al., 2015). A large meta‐analysis revealed systolic and diastolic BP are each reduced by ~5 mm Hg with HIIT in overweight and obese cohorts (Batacan et al., 2017). Furthermore, HIIT is an effective therapy for improving glycemic control (Babraj et al., 2009; Batacan et al., 2017; Little et al., 2011; Richards et al., 2010). However, no study has investigated the effects of HIIT predominantly using the upper limbs in patients with osteoarthritis.

A second treatment proposed here is passive heat therapy – gaining interest as a partial exercise mimetic due to its beneficial effect on cardiovascular health and association with reduced all‐cause mortality (Cullen et al., 2020; Laukkanen et al., 2015). Passive heat therapy is in fact not novel but centuries old, historically used as a form of healing to treat health conditions such as rheumatism and skin conditions (Fagan, 2006; Lehtmets, 1957; Nicholls & Harwood, 2017). While passive heat therapy cannot mimic all of the beneficial effects of exercise, it does appear to have a greater anti‐hypertensive effect than exercise (Akerman et al., 2019), has *no* impact on the joints, and may therefore be an appropriate treatment for those with osteoarthritis. In a recent trial in our laboratory, resting systolic and diastolic BP decreased 7 mmHg and 4 mmHg following 12 weeks of heat therapy (3–5 × weeks, 20–30 min immersion in 40°C water) in patients with peripheral arterial disease (Akerman et al., 2019); this was double the reduction observed with supervised exercise. These adaptive hypotensive effects have also been reported in healthy and other clinical populations (Brunt et al., 2016; Ely, Francisco, et al., 2019; Hoekstra et al., 2018). With respect to glycemic control, studies in sedentary and clinical populations have reported improvements with regular hot‐water immersion (Ely, Clayton, et al., 2019; Ely, Francisco, et al., 2019; Hoekstra et al., 2018; Hooper, 1999). Despite these promising findings, study sample sizes investigating the anti‐hypertensive effects of heat therapy have been low (≤11 participants) and were undertaken in select cohorts with specific cardiovascular disease risk profiles (Akerman et al., 2019; Brunt et al., 2016; Ely, Francisco, et al., 2019). Osteoarthritis is associated with autonomic dysfunction that not only contributes to progression of the disease (Yeater et al., 2022; Yeater et al., 2023), but may also elicit divergent cardiovascular and metabolic responses from previously reported cohorts (Bossenger et al., 2023; Yeater et al., 2023).

The overall aim of this study was to investigate the acute and adaptive cardiovascular and metabolic effects of heat therapy and upper‐limb HIIT, and to determine if acute BP responses were predictive of adaptive changes in BP in patients with severe lower‐limb osteoarthritis. The study hypotheses were: (1) acute hot‐water immersion and HIIT exposure would elicit post‐exposure hypotension (2) acute reductions in BP during hot‐water immersion exposure would not be different after 12 weeks of repeated exposure; (3) repeated hot‐water immersion and HIIT exposure would reduce resting blood pressure across 12 weeks of repeated exposure; and (4) the magnitude of BP reduction following acute hot‐water immersion or HIIT exposure would be associated with adaptive changes in resting BP after 12 weeks of repeated exposure.

## 2 METHODS

### 2.1 Ethics statement

Ethical approval for the study was obtained from the Health and Disability Ethics Committee of New Zealand (Ref: 18/NTA/148) and the study was registered with the Australia New Zealand Clinical Trial Registry (ACTRN12618001358235). Written, informed consent was obtained for all participants, and all procedures conformed to the standards set by the Declaration of Helsinki.

### 2.2 Experimental design

This is a sub‐study of a multi‐arm, parallel design, randomized, controlled trial, for which objectives, methods, and results for the primary outcome variable is described elsewhere (under review). This present analysis includes participants who were randomized to either passive heat therapy (Heat) or high‐intensity interval training (HIIT) in the original randomized controlled trial. To compare the effects of the intervention to an active control group, data from the home‐based exercise (Home) group are also presented; as we did not assess the acute effects of the Home exposure, those data are not presented.

**FIGURE 1:**
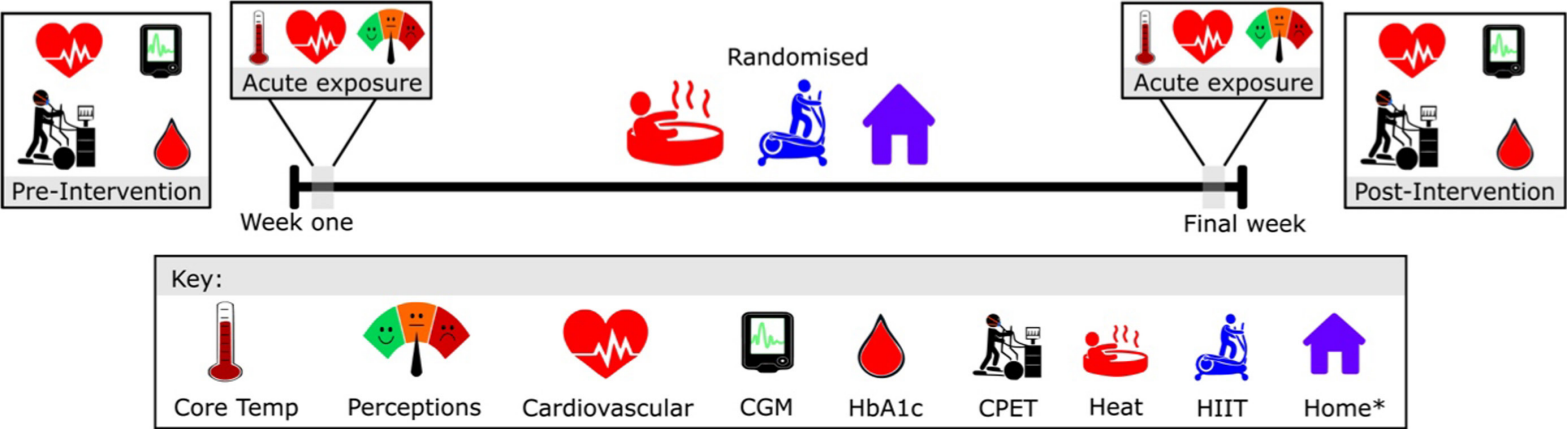
Schematic representation of acute (week one and final week) and adaptive (pre‐intervention and post‐intervention) experimental measures. CGM, continuous glucose monitoring; CPET, cardiopulmonary exercise test; HbA1c, glycated hemoglobin; Heat, hot‐water immersion/passive heat therapy; HIIT, high‐intensity interval training; Home, home‐based exercise. *Acute exposure not assessed.

Eligible, consenting, and screened participants underwent an initial baseline assessment prior to randomization (Figure 1). Participants then attended their initial Heat or HIIT intervention session and physiological and psychophysical measures were collected to characterize the initial acute exposure. These measurements were repeated during exposure in the final week of the intervention, and measurements performed during the baseline assessment session were repeated following completion of the intervention.

### 2.3 Study participants

Patients with end‐stage hip or knee osteoarthritis and waitlisted for hip/knee arthroplasty at Dunedin Public Hospital (Dunedin, New Zealand) were recruited for this study. Participants were excluded if they met any of the following criteria: (1) a contraindication to non‐physician supervised maximal exercise testing (Fletcher et al., 2013); (2) stable or unstable angina; (3) a recent myocardial infarction (i.e., < 3 months ago); (4) an implantable cardioverter defibrillator or pacemaker; (5) revision arthroplasty; (6) staged bilateral total joint replacement; (7) pathology limiting upper‐limb exercise (i.e., shoulder‐joint osteoarthritis); and (8) any other medical condition deemed by the study anesthetist/cardiologist as a significant risk to study participation.

### 2.4 Randomization and blinding

Participants were randomly assigned to intervention groups using a concealed randomization process, using computer‐generated permuted block randomization, with random block size and stratification for site of scheduled arthroplasty (i.e., hip or knee) (Urbaniak & Plous, 2013).

### 2.5 Interventions

#### 2.5.1 Passive heat therapy (Heat)

The protocol was based on previous research from our laboratory (Akerman et al., 2019), and literature that demonstrated increases in peak $\dot{V}$O_2_ (Bailey et al., 2016; Hesketh et al., 2019). Participants completed three hot‐water immersion sessions per week for 12 weeks (less if surgery scheduled prior), with bathing duration progressively increasing from 20 to 30 min in weeks 2–3, as tolerable. Participants were seated in swimming attire in a temperature‐controlled spa (week one: 39.9 ± 0.3°C; final week: 40.0 ± 0.3°C) with water approximately mid‐sternal level. The room was temperature controlled (~22.2°C and ~24% relative humidity). At the end of hot‐water immersion, participants exited the pool, dried themselves, and dressed in comfortable clothing for exercise. Participants then completed 10–20 min of light‐intensity resistance exercise (calisthenics). Participants performed 10 exercises, progressing to 12–20 reps of each (Pescastello et al., 2013). Participants were recommended to alternate between upper‐body and lower‐body exercises, to ensure adequate recovery between sets. Elastic bands of varying resistance were used, with progression as exercise tolerance increased. *Note*: *for the initial session only, exposure duration was 20 min followed by 20 min seated rest* (i.e., *no calisthenics*).

#### 2.5.2 High‐intensity interval training (HIIT)

Participants performed the exercise on either a cross‐trainer (NordicTrack e12.2) or arm ergometer (Schwinn Windjammer) (dependent on original exercise test modality, dictated by their capability/pain). A familiarization session was performed prior to the initial acute exposure session to determine an individualized starting exercise intensity according to a protocol described by Phillips et al. (2017). Following a 3–5 min warm‐up, initial sessions began with 6 × 60‐s intervals, separated by 90‐s active recovery (very‐light intensity); duration of exercise intervals remained constant throughout the intervention, with the number of intervals increasing to 8, and recovery duration decreasing to 60 s, based on participant progress. To account for the anticipated increase in peak $\dot{V}$O_2_ throughout the study and to maintain protocol progression, exercise intensity was periodically increased to ensure a rating of perceived exertion of 7/10 (very hard) but less than 90% heart rate reserve was maintained. Each session lasted approximately 20 min and included a cool‐down with whole‐body flexibility exercises.

#### 2.5.3 Home‐based exercise (Home)

In addition to standard care (advice to eat well and stay active throughout their waiting period), participants were given a resistance band and a home‐based exercise program. The exercise program consisted of the same exercises performed by Heat participants aimed at increasing muscle strength and flexibility. Participants were encouraged to perform the program 3 days per week for ~15–20 min. Participants were asked to record sessions in an activity diary provided to them. A pragmatic decision was made to include an active control group as participants in this study were scheduled for surgery and are highly motivated to exercise; in our experience, patients who are randomized to standard care *only* are highly likely to drop out or perform their own independent exercise. Hence, the decision was made to offer these participants support in the form of an intervention that may aid their recovery from surgery (e.g., ambulating with crutches) without affecting our primary outcome measure.

### 2.6 Experimental procedures

Assessment sessions were conducted at similar times of the day for each participant and these sessions were at least 1 day apart from each other. Participants were asked to follow a standardized diet and activity regimen prior to each assessment session. This included abstention from cigarette smoking 4 h prior, alcohol and caffeinated beverages 12 h prior, and moderate‐ or high‐intensity exercise for at least 24 h prior. Participants were advised to avoid any non‐ambient heat exposure (e.g., sauna use) outside of prescribed sessions and reminded to continue taking their normal medications. All measurements were performed by the same experienced researcher (BR). Testing was performed in a climate‐controlled room to maintain an appropriate ambient temperature (20–22°C) and humidity (<60% relative humidity).

### 2.7 Characterizing acute exposure

A clinical exercise physiologist supervised all Heat and HIIT sessions, recording attendance, compliance and pre‐, intra‐, and post‐session BP and heart rate, as well as pain (0–10, no pain–worst possible pain), perceived exertion (0–10, nothing at all–maximal), thermal sensation (1–13, unbearably cold–unbearably hot), and discomfort (1–10, comfortable–extremely uncomfortable) using validated scales (Bijur et al., 2001; Borg, 1982; Gagge et al., 1967). Measurements were performed during acute exposures in the first and final week of the intervention. Participants were seated for at least 5 min and resting BP measured using a sphygmomanometer and appropriately sized cuff (Welch Allyn DS66) on the left arm twice and averaged; a third measure was performed if systolic or diastolic BP differed by ≥5 mm Hg, and a median calculated. Resting heart rate was obtained and during exposure was continuously recorded every 5 s (RS800). BP, heart rate, and psychophysical measures were obtained every 10 min during Heat exposure and at the end of the final exercise interval during HIIT exposure. Following exposure, seated BP and heart rate were measured at 5‐min intervals for 20 min (first session only). Rate pressure product was calculated to estimate myocardial oxygen demand as the product of systolic BP and heart rate.

To quantify the thermal stimulus, body core temperature was measured in a subset (*n* = 10 per group) of participants during the first and final week of each intervention (a session that included calisthenics). An ambulatory monitoring system (Bodycap) continuously recorded body core temperature from an ingestible pill thermometer (ingested 3–4 h prior to recording).

### 2.8 Characterizing changes across the intervention

To characterize the adaptive effect of the interventions (i.e., across 12 weeks), all participants completed measurements of resting BP, heart rate, and heart rate variability. A baseline assessment was performed prior to randomization and repeated at least 1 day following the final session of the intervention.

Resting BP was collected on the left arm per American Heart Association guidelines (Muntner et al., 2019). In brief, participants lay supine for at least 10 min and measurement was completed twice and averaged; a third measure was performed if systolic and/or diastolic BP differed by ≥5 mm Hg, and a median calculated.

Heart rate variability (HRV) was assessed using a 3‐lead electrocardiogram (Lead II position; FE132, ADInstruments) to measure heart rate and rhythm from the R‐R intervals of ventricular depolarization. Once connected to the electrocardiogram, each participant rested in a supine position for at least 5 min, before collecting HRV data for at least 5 min (Electrophysiology task force of the European Society of Cardiology the North American Society of Pacing, 1996). Resting heart rate was calculated as the average heart rate over the five‐minute monitoring period.

Glycated hemoglobin (HbA1c) was analyzed via a whole blood sample (D‐100 HbA_1c_ System, Bio‐Rad) taken during the cardiovascular assessment session. Continuous blood glucose monitoring (CGM; Freestyle Libre Pro, Abbot) was performed in a subset of participants (*n* = 10 per group); interstitial glucose concentration was recorded at 15‐min intervals over at least a 7‐day period; a food diary was kept over the monitoring period and participants were asked to replicate the diet during post‐intervention assessment.

### 2.9 Data analysis

Daily average CGM was calculated as the mean glucose across all valid wear days (i.e., days with a full 24 h of data). Night average was calculated as the mean glucose across all valid wear days between 10 p.m. and 6 a.m. Time in range was considered where glucose was between 3.9 and 10.0 mmol L^−1^. Glucose management indicator is a novel measure for estimating A1c from CGM devices (Bergenstal et al., 2018); this was calculated as 12.71 + 4.70587 × [mean glucose in mmol L^−1^]. Area under the curve was calculated using the trapezoidal rule.

### 2.10 Statistical analysis

A sample size calculation was performed for the primary outcome variable for the overall trial and is detailed elsewhere (under review). All statistical analyses were performed using SPSS (v27, IBM) and graphed using Prism (v9.3, GraphPad). Descriptive data were expressed as raw mean (±SD), median (IQR) or number (proportion). Comparisons of interest are reported as model adjusted estimated marginal means with 95% confidence interval [lower limit, upper limit]. A mixed model analysis of variance (ANOVA) tested for between and within group differences during acute exposures. A mixed model analysis of covariance (ANCOVA), using number of intervention sessions as the covariate tested for significant between‐group differences in baseline and end of intervention cardiovascular and glycemic variables. Homogeneity of variances were assessed with Levene's tests. A Bonferroni adjustment was performed to account for multiple comparisons. A check for normality of residuals was performed for each variable by visually inspecting Q‐Q plots and assessed formally using a Shapiro–Wilk test; where normality was violated for a variable, raw data were log transformed. A Tukey post hoc test was performed if statistical significance was observed to elucidate differences between groups. Ordinal data were analyzed using the Mann–Whitney *U* or Friedman tests, with Wilcoxon signed rank tests used to isolate differences for the later. Pearson correlation coefficients and simple linear regression assessed the relationship between the post‐exposure BP response following an acute bout of hot‐water immersion and HIIT, and the adaptive change in BP across the intervention. To aid in the interpretation of the correlation coefficients, 0.9–1.0 was considered a very high correlation, 0.70–0.90 high correlation, 0.5–0.7 moderate correlation, 0.3–0.5 low correlation and 0.0–0.30 negligible correlation (Mukaka, 2012). A significance level of 0.05 was used for all tests.

## 3 RESULTS

### 3.1 Participant characteristics

Ninety‐three eligible participants were recruited and completed baseline assessments. Participants' descriptive characteristics are presented in Table 1. Two Heat participants were excluded from the intervention BP analysis due to a change in anti‐hypertensive medication across the course of the intervention. One Heat participant was excluded from acute exposure analysis due to technical issues with the measurement.

**TABLE 1:** Descriptive statistics of participants.

Variable	Heat (*n* = 27)	HIIT (*n* = 25)	Home (*n* = 26)
Age (years)	66 (7)	71 (9)	67 (8)
Male/female	13 (48%)/14 (52%)	13 (52%)/12 (48%)	11 (42%/15 (58%)
BMI (kg^.^m^−2^)	31.6 (6.6)	32.2 (5.3)	32.0 (8.0)
Arthroplasty site			
Hip	11 (41%)	11 (44%)	11 (42%)
Knee	16 (59%)	14 (56%)	15 (58%)
Comorbidity			
Asthma/COPD	7 (26%)	5 (20%)	5 (19%)
CVD			
Previous myocardial infarct	5 (19%)	3 (12%)	0
Atrial arrhythmia	0	1 (4%)	2 (8%)
Previous stroke	3 (11%)	1 (4%)	0
Dyslipidemia	13 (48%)	11 (44%)	7 (27%)
Hypertension	18 (67%)	17 (68%)	14 (54%)
Obesity	15 (56%)	12 (48%)	14 (54%)
Diabetes mellitus/pre‐diabetes	8 (30%)	4 (16%)	4 (15%)
Medication			
ACE inhibitor	9 (33%)	4 (16%)	11 (42%)
ARB	8 (30%)	6 (24%)	2 (8%)
Beta‐adrenergic blocker	8 (30%)	6 (24%)	2 (8%)
Calcium channel blocker	5 (19%)	8 (32%)	6 (23%)
Diuretic	6 (22%)	3 (12%)	8 (31%)
Statin	14 (52%)	12 (48%)	7 (27%)
Anti‐glycemic agent	6 (22%)	3 (12%)	2 (8%)
Insulin	3 (11%)	0	1 (4%)
Reported PA status			
No physical activity	11 (41%)	10 (40%)	9 (35%)
Active, but not meeting PA guidelines	5 (19%)	5 (20%)	6 (23%)
Meeting PA guidelines	11 (41%)	10 (40%)	11 (42%)

*Note*: Data are mean (SD), median (IQR) or as an absolute number with the percentage (%) of the whole.

Abbreviations: ACE, angiotensin‐converting‐enzyme; ARB, angiotensin receptor blockers; BMI, body mass index; COPD, chronic obstructive pulmonary disease; CVD, cardiovascular disease; PA, physical activity; anti‐glycemic agents include metformin, SGLT‐2 inhibitors, sulfonylureas and DPP‐4 inhibitors.

### 3.2 Characterizing the acute exposure

#### 3.2.1 Systolic blood pressure

**FIGURE 2:**
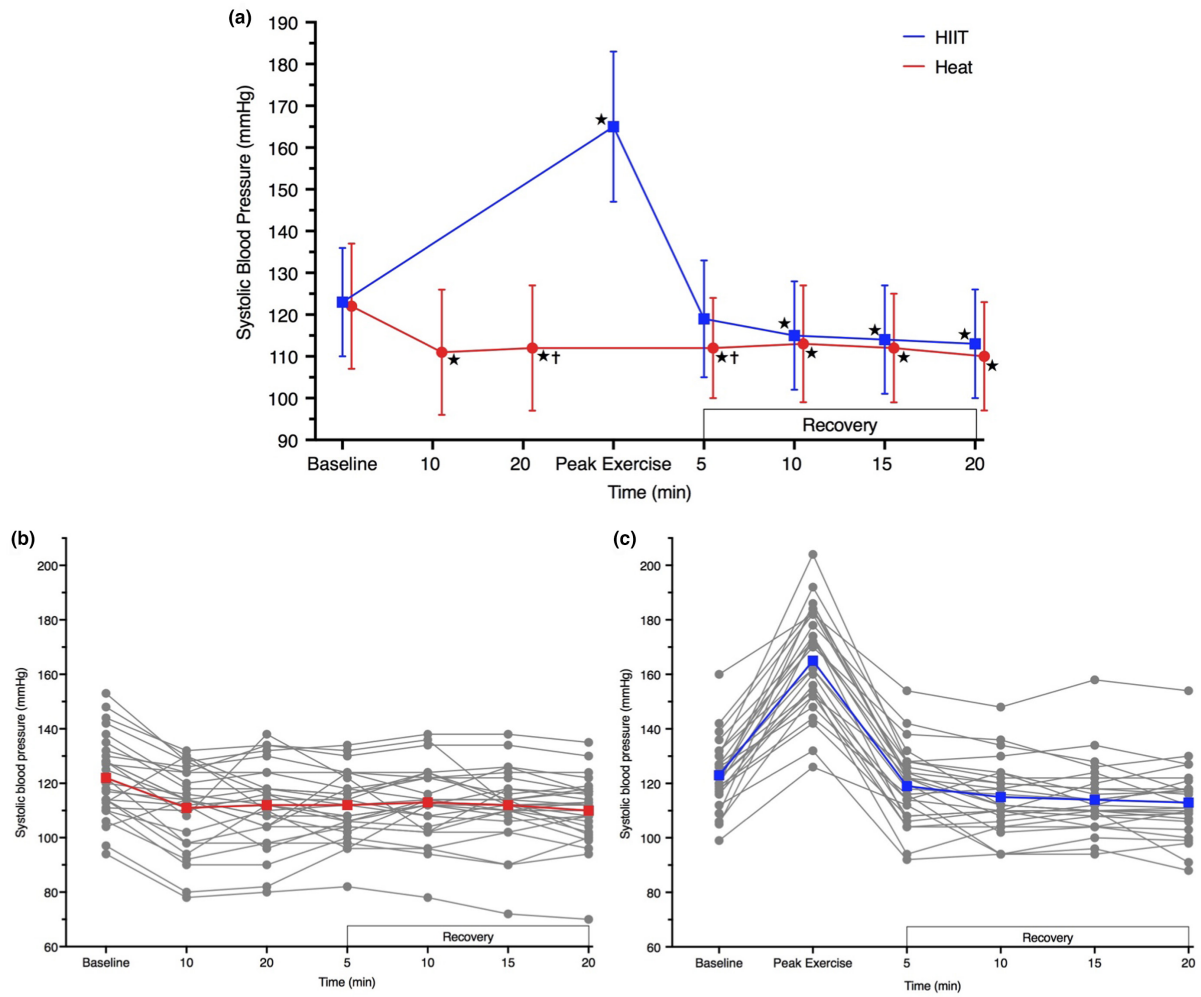
Systolic blood pressure during an acute bout of hot‐water immersion and high‐intensity interval training and 20 min seated recovery (a), during session one. Individual responses are presented for hot‐water immersion (b) and HIIT (c) participants. Data were analyzed using a mixed model ANOVA test. *p < 0.05 versus baseline; ^†^ *p* < 0.05 versus HIIT. Peak exercise measure obtained at end of the final interval. *n* = 26 for Heat and *n* = 25 for HIIT.

By 10 min of hot‐water immersion, systolic BP was 11 mm Hg lower ([−16, −5], *p* < 0.001) than baseline; it remained 10–12 mm Hg lower during immersion and throughout 20 min of seated rest after immersion (all *p* < 0.001 vs. baseline; Figure 2 and Table S1). During an acute HIIT bout, systolic BP was 42 mm Hg higher ([32, 52], *p* < 0.001) than baseline, but by 10 min post‐exposure, and thereafter, it was 8–10 mm Hg lower than baseline (all *p* < 0.001 vs. baseline; Figure 2 and Table S2). Only the stress period itself and the first 5 min after exposure was different between groups (*p* = 0.013; all other recovery time points were not different, *p* ≥ 0.168).

Between the first and last week of the intervention at comparable hot‐water immersion exposure time points (i.e., 10‐ and 20‐min immersion), there was no difference in systolic BP (*p* ≥ 0.468; Table S2). Systolic BP at the end of immersion in the last week of the intervention (i.e., 30 min immersion) was lower than at the end of immersion in session one (vs. session one: 20 min; −8 mm Hg [−14, −3], *p* = 0.004); indicating the extra 10 min hot‐water immersion had an additional hypotensive effect.

#### 3.2.2 Diastolic blood pressure

**FIGURE 3:**
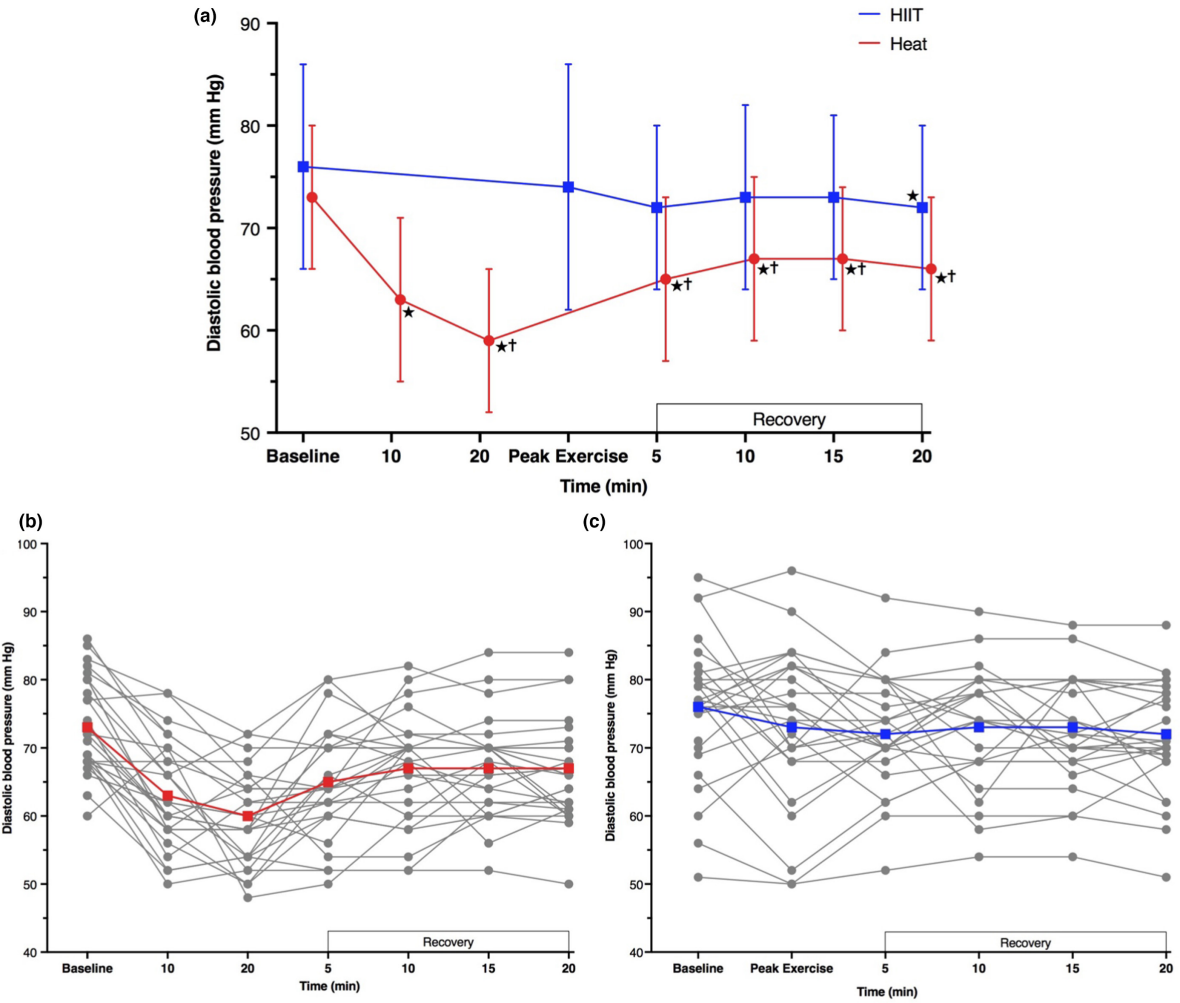
Diastolic blood pressure during an acute bout of hot‐water immersion and high‐intensity interval training and 20 min seated recovery (a), during session one. Individual responses are presented for hot‐water immersion (b) and HIIT (c) participants. Data were analyzed using a mixed model ANOVA test. *p < 0.05 versus baseline; ^†^ *p* < 0.05 versus HIIT. Peak exercise measure obtained at end of the final interval. *n* = 26 for Heat and *n* = 25 for HIIT.

At 20 min of hot‐water immersion, diastolic BP was 14 mm Hg lower ([−18, −10], *p* < 0.001) than baseline (Figure 3). Diastolic BP increased during the first 10 min of recovery (vs. 20 min: +7 mm Hg [3, 11], *p* < 0.001) and after 20 min of recovery remained 7 mm Hg lower than baseline ([−10, −3], *p* < 0.001). During an acute HIIT bout, exercise diastolic BP was not different from baseline and only after 20 min of seated rest was it lower than baseline (−4 mm Hg [−8, 0], *p* = 0.026). Except baseline (*p* = 0.171), diastolic BP was lower at all time points during hot‐water immersion, compared to HIIT (*p* ≤ 0.009).

Between the first and last week of the intervention at comparable hot‐water immersion exposure time points (i.e., 10 and 20 min immersion), there was no difference in diastolic BP (*p* ≥ 0.214); however, diastolic BP was lower at the end of immersion (i.e., 30 min) in the final week, compared to session one (vs. session one: 20 min; −4 mm Hg [−8, −1], *p* = 0.006).

#### 3.2.3 Mean arterial blood pressure

**FIGURE 4:**
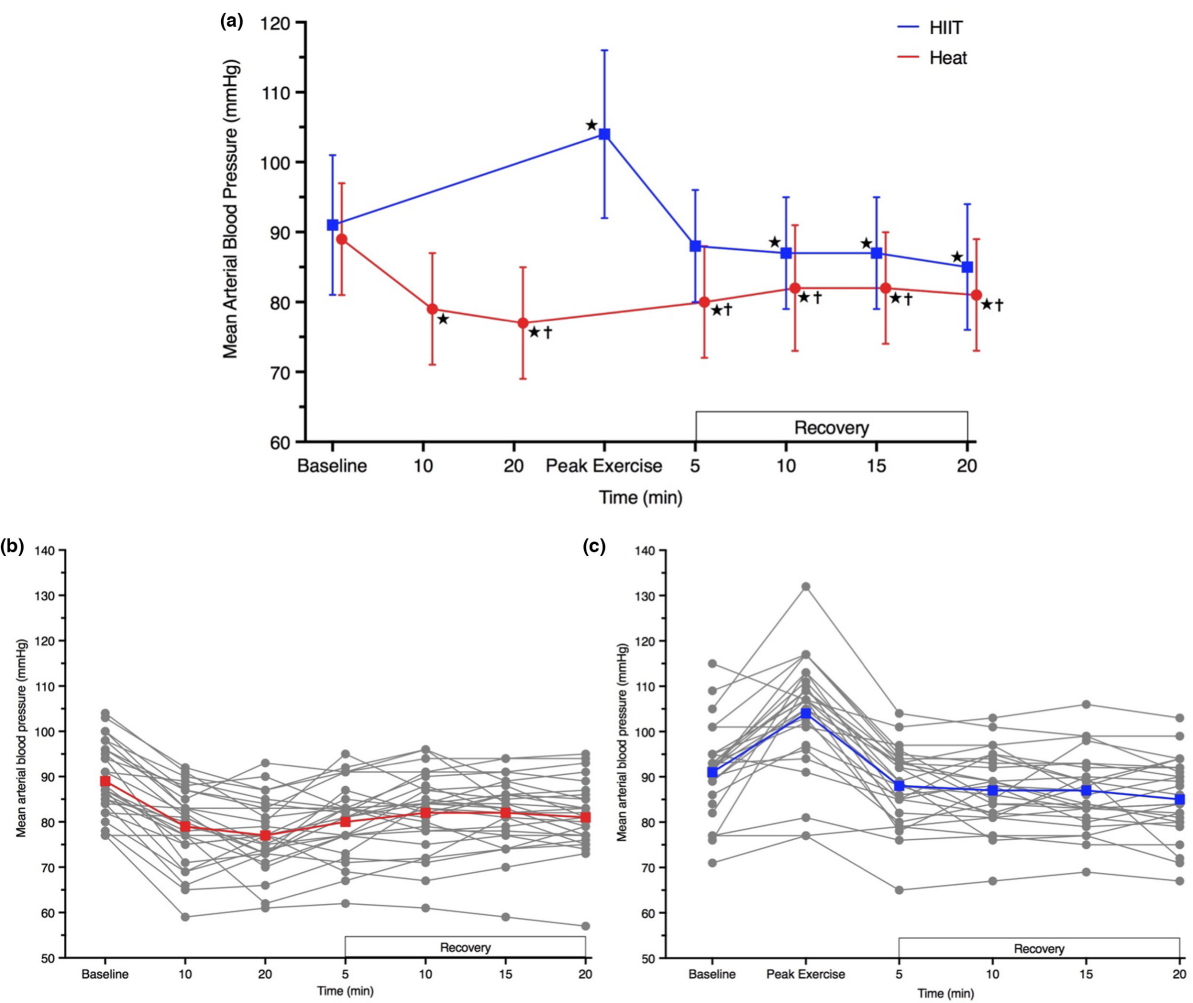
Mean arterial blood pressure during an acute bout of hot‐water immersion and high‐intensity interval training and 20 min seated recovery (a), during session one. Individual responses are presented for hot‐water immersion (b) and HIIT (c) participants. Data were analyzed using a mixed model ANOVA test. *p < 0.05 versus baseline; ^†^ *p* < 0.05 versus HIIT. Peak exercise measure obtained at end of the final interval. *n* = 26 for Heat and *n* = 25 for HIIT.

After 20 min of hot‐water immersion mean arterial BP was 13 mm Hg lower ([−16, −9], *p* < 0.001) than baseline (Figure 4) and remained 8 mm Hg lower than baseline post‐immersion ([−11, −5], *p* < 0.001). During an acute HIIT bout, exercise mean arterial BP was 12 mm Hg higher ([6, 18], *p* < 0.001) than baseline (Figure 4). Following 10 and 20 min of seated rest post‐HIIT, mean arterial BP remained lower than baseline (−4 mm Hg [−8, −1], *p* = 0.010; −6 mm Hg [−10, −3], *p* < 0.001).

#### 3.2.4 Heart rate

**FIGURE 5:**
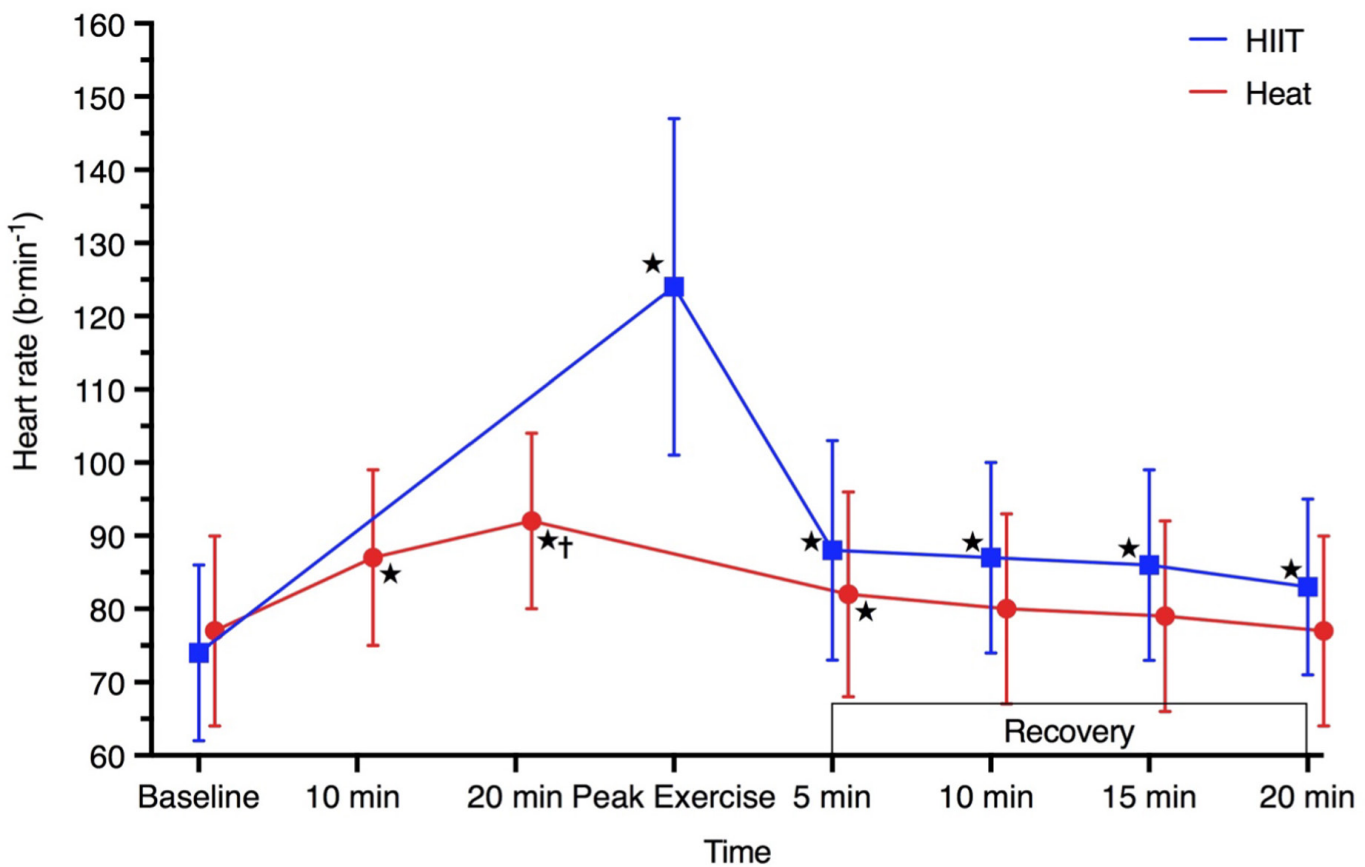
Heart rate during an acute bout of hot‐water immersion and high‐intensity interval training and 20 min seated recovery, during session one. Data are exposure mean (error bars indicate SD) and were analyzed using a mixed model ANOVA test. *p < 0.05 versus baseline; ^†^ *p* < 0.05 versus HIIT. Peak exercise measure obtained at end of the final interval. *n* = 26 for Heat and *n* = 25 for HIIT.

After 10 and 20 min of hot‐water immersion, heart rate was 11 b·min^−1^ higher ([6, 15], *p* < 0.001) and 16 b·min^−1^ higher ([11, 20], *p* < 0.001) than baseline (Figure 5) but was not different after 10 min of recovery (*p* ≥ 0.064). During an acute HIIT bout, peak‐exercise heart rate was 50 b·min^−1^ higher ([39, 62], *p* < 0.001) than baseline and remained 9 b·min^−1^ higher after 20 min of rest ([3, 14], *p* = 0.001). Heart rate was statistically higher in HIIT than Heat only during exposure (*p* < 0.001).

**FIGURE 6:**
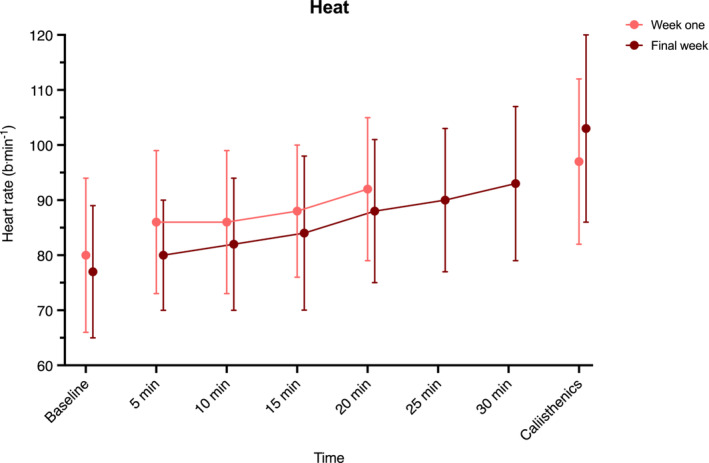
Heart rate response during hot‐water immersion and after calisthenics, in week one (20 min exposure) and final week of the intervention (30 min exposure). Data are an average across each interval/recovery period and presented as mean (error bars indicate SD) and were analyzed using a mixed model ANOVA test. *n* = 25.

**FIGURE 7:**
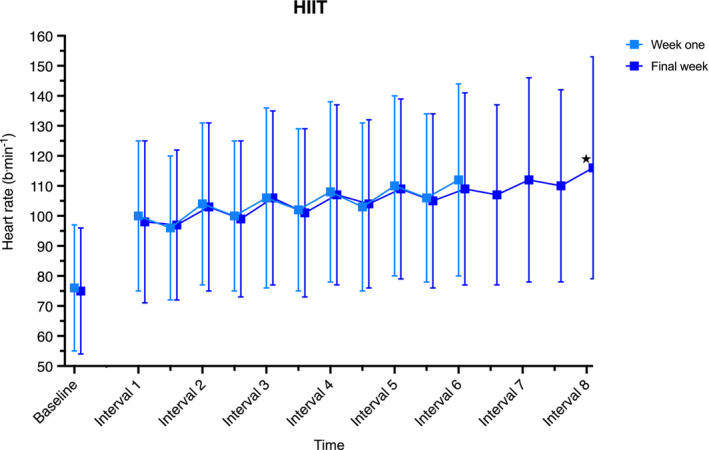
Heart rate response during high‐intensity interval training, in week one (6 × intervals) and final week of the intervention (8 × intervals). Data are an average across each interval/recovery period and presented as mean (error bars indicate SD) and were analyzed using a mixed model ANOVA test. *p < 0.05 week one, interval 6 versus final week, interval 8. *n* = 25.

Heart rate in the Heat group was not different in the final week compared to first week of the intervention at 20 min immersion, end of exposure or end of calisthenics (*p* ≥ 0.079; Figure 6). During HIIT, average heart rate across the final interval was 7 b·min^−1^ higher ([1, 14], *p* = 0.032) in the final week compared to first week of the intervention (Figure 7).

#### 3.2.5 Rate pressure product

In week one, rate pressure product during the final high‐intensity interval was more than double that after 20 min hot‐water immersion (21,198 ± 4521 vs. 10,266 ± 2092 mm Hg b·min^−1^; *p* < 0.001). For Heat participants, rate pressure product was not different at 20‐min immersion in week one (9507 b mm Hg·min^−1^ [8594, 10,419]) and 30‐min immersion in the final week of the intervention (9869 b mm Hg·min^−1^ [8692, 11,046]; *p* = 0.142).

#### 3.2.6 Body core temperature

**FIGURE 8:**
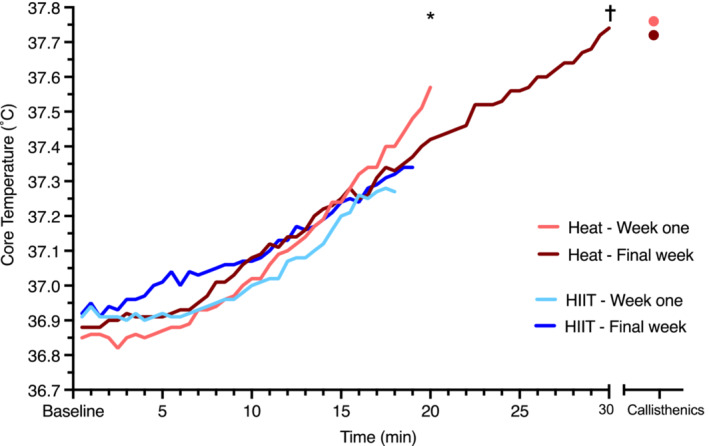
Body core temperature response during an acute bout of hot‐water immersion and after calisthenics, and during high‐intensity interval training in week one and the final week of the intervention. Data are exposure mean and were analyzed using a mixed model ANOVA test. *p < 0.05 hot‐water immersion versus HIIT at end of exposure in week one; ^†^ *p* < 0.05 versus hot‐water immersion versus HIIT at end of exposure in the final week of intervention. *n* = 9 for Heat and *n* = 8 for HIIT.

In week one, body core temperature increased during water immersion by 0.9°C [0.6, 1.2] and remained elevated by 0.9°C [0.7, 1.1] at completion of calisthenics (Figure 8); this increase was not different in the final week of the intervention (*p* ≥ 0.593). Core temperature rose 0.4°C [0.1, 0.7] during HIIT in both week one and the final week of the intervention. Body core temperature therefore increased 0.5°C more ([0.1, 0.9], *p* = 0.018) with hot‐water immersion than HIIT during week one, and 0.4°C more ([0.1, 0.8], *p* = 0.029) in the final week of the intervention.

#### 3.2.7 Psychophysical

At the end of hot‐water immersion in week one, median thermal sensation was 10 AU (i.e., “Hot”; [9, 10]) and thermal discomfort 2 AU (i.e., comfortable – slightly uncomfortable; [1, 4]). Thermal sensation was higher at the end of hot‐water immersion in the final week of the intervention (10 AU [10, 11]; *p* < 0.001), but thermal discomfort was not (2 AU [1, 4], *p* = 0.080), compared to week one. Median rating of perceived exertion during HIIT in week one was 6 AU (i.e., hard – very hard; [5, 7]) and was not different in the final week of the intervention (7 AU [6, 7], *p* = 0.176).

### 3.3 Characterizing the effect of the interventions

Compliance to the treatment was not different between groups (*p* = 0.312); Heat, HIIT, and Home participants completed 36 ± 11, 33 ± 11 and 40 ± 23 sessions, respectively. Home participants reported performing a median ~20 reps for 1 set per session; this was not different to calisthenics performed in the Heat group (16 reps, and 1 set; *p* ≥ 0.851). Sixteen HIIT participants completed training on the arm ergometer and nine on the cross trainer.

#### 3.3.1 Resting blood pressure

**FIGURE 9:**
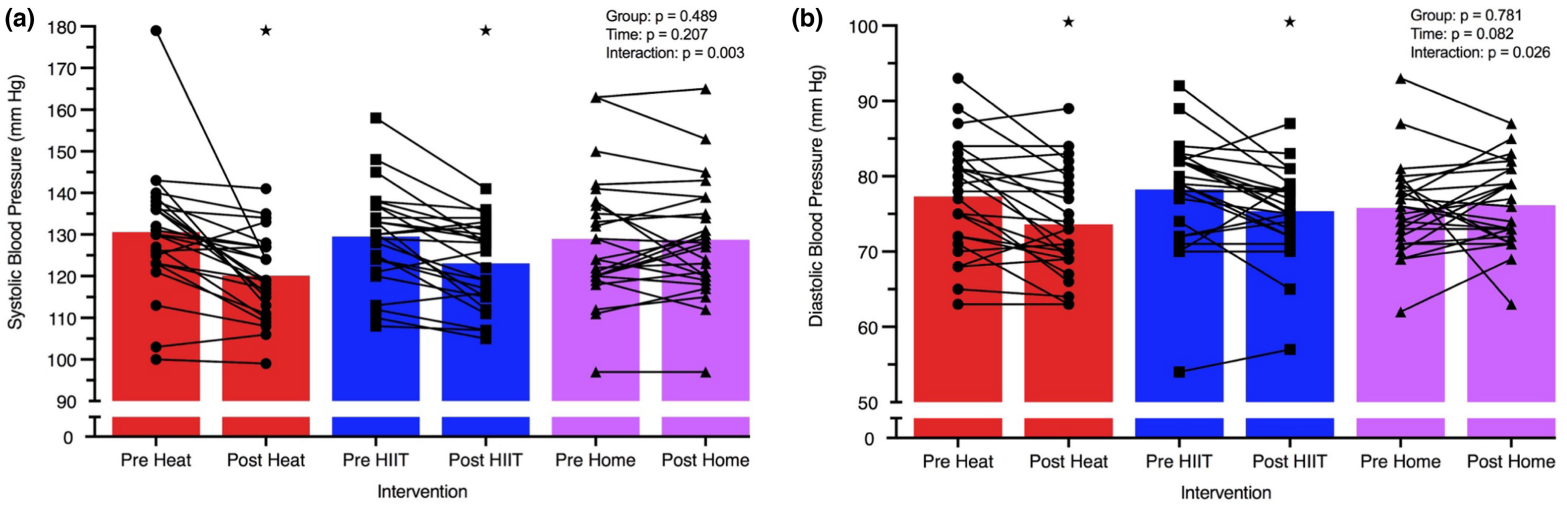
Pre‐ and post‐intervention resting systolic (a) and diastolic (b) blood pressure for Heat, HIIT, and Home groups. Results from individual participants (lines represent change from pre‐ to post‐intervention) and modality mean (colored bars) are presented for each intervention. Differences in means were analyzed using a mixed model ANOVA test; *p < 0.05 versus pre‐intervention. *N* = 25 for Heat, *n* = 25 for HIIT and *n* = 26 for Home.

Reductions in systolic BP and diastolic BP were evident in both Heat (systolic BP: −9 mm Hg [−13, −5], *p* < 0.001; diastolic BP: −4 mm Hg [−6, −2], *p* ≤ 0.001) and HIIT (−7 mm Hg [−11, −3], *p* = 0.001; −3 mm Hg [−5, −1], *p* = 0.011; Table 2, Figure 9). Accordingly, mean arterial BP was reduced by 6 mm Hg and 4 mm Hg with both Heat and HIIT (both *p* < 0.001). Systolic (0 mm Hg [−4, 4]) diastolic (0 mm Hg [−2, 2]) and mean arterial BP (0 mm Hg [−2, 2]) remained unchanged with Home (*p* ≥ 0.785). Systolic BP was reduced to a greater extent with Heat (vs. Home: −9 mm Hg [−15, −3], *p* = 0.004) and HIIT (vs. Home: −6 mm Hg [−12, −1], *p* = 0.049), and mean arterial BP to a greater extent with Heat (vs. Home: −4 mm Hg [−8, −1], *p* = 0.021); no other between group differences were observed (*p* ≥ 0.161). In a sub‐analysis of patients with hypertension (i.e., taking at least one antihypertensive medication; *n* = 17), there was a greater decrease in systolic BP (−15 mm Hg [−20, −9]) and diastolic BP (−5 mm Hg [−8, −2]) across the Heat intervention compared with participants without hypertension (SBP: −1 mm Hg [−9, +6], interaction: *p* = 0.002; DBP: −1 mmHg [−4, +3], interaction: *p* = 0.002; *n* = 10). No differences were observed with HIIT (*p* = 0.323).

**TABLE 2:** Resting cardiovascular indices in Heat, HIIT, and Home groups.

Variable	Heat		HIIT		Home		Statistical significance		
	PRE	POST	PRE	POST	PRE	POST	Group	Time	Interaction
Resting blood pressure									
SBP (mm Hg)	130 (15)	120 (10)*	130 (12)	123 (10)*	129 (15)	129 (14)	0.489	0.207	0.003
DBP (mm Hg)	77 (7)	74 (7)*	78 (7)	75 (6)*	76 (6)	76 (6)	0.781	0.082	0.026
MAP (mm Hg)	95 (9)	89 (7)*	95 (8)	91 (6)*	94 (8)	94 (7)	0.690	0.067	0.001
Heart rate variability									
Resting heart rate (b·min^−1^)	68 (9)	69 (9)	68 (10)	67 (11)	65 (10)	68 (11)	0.711	0.569	0.273
Total power	719 (605)	785 (872)	597 (450)	608 (449)	728 (297)	669 (466)	0.469	0.502	0.567
Low‐frequency power	191 (194)	230 (308)	148 (141)	143 (129)	197 (113)	211 (184)	0.162	0.718	0.810
High‐frequency power^a^	86 (88)	104 (129)	146 (177)	163 (195)	145 (86)	100 (87)	0.157	0.389	0.195
LF/HF ratio^a^	2.8 (2.7)	2.5 (1.7)	1.9 (2.0)	1.6 (2.3)	1.9 (1.2)	2.7 (2.0)	0.200	0.898	0.184

*Note*: Variables are presented as mean (SD) and analyzed with a mixed model ANOVA.

Abbreviations: DBP, diastolic blood pressure; LF/HF ratio, low‐frequency power/high‐frequency power ratio; MAP, mean arterial pressure; POST, post‐intervention; PRE, pre‐intervention; SBP, systolic blood pressure.

^a^ Log transformed for statistical analysis.

* *p* < 0.05 versus PRE.

#### 3.3.2 Resting heart rate and heart rate variability

Resting heart rate (*p* = 0.273) and HRV domains (*p* ≥ 0.195; Table 2) were unchanged with either intervention.

#### 3.3.3 Predictive value of acute blood pressure response

**FIGURE 10:**
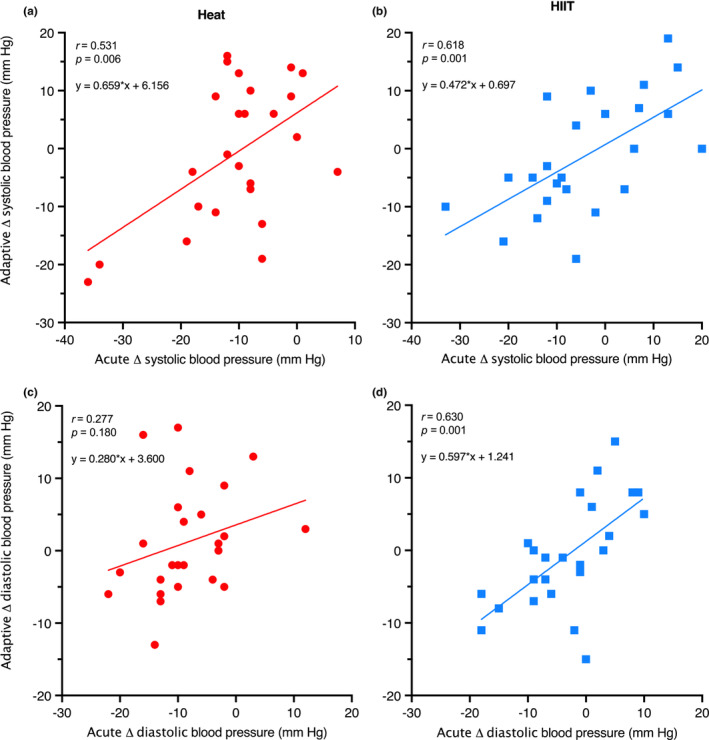
The magnitude of change (Δ) between pre‐session and 5‐min post‐session systolic (a & b – top graphs) and diastolic BP (c & d – bottom graphs) (acute; *x*‐axis), and the magnitude of change (Δ) in resting systolic BP and diastolic BP across the intervention (adaptive; *y*‐axis), for Heat (red) and HIIT (blue) participants.

**FIGURE 11:**
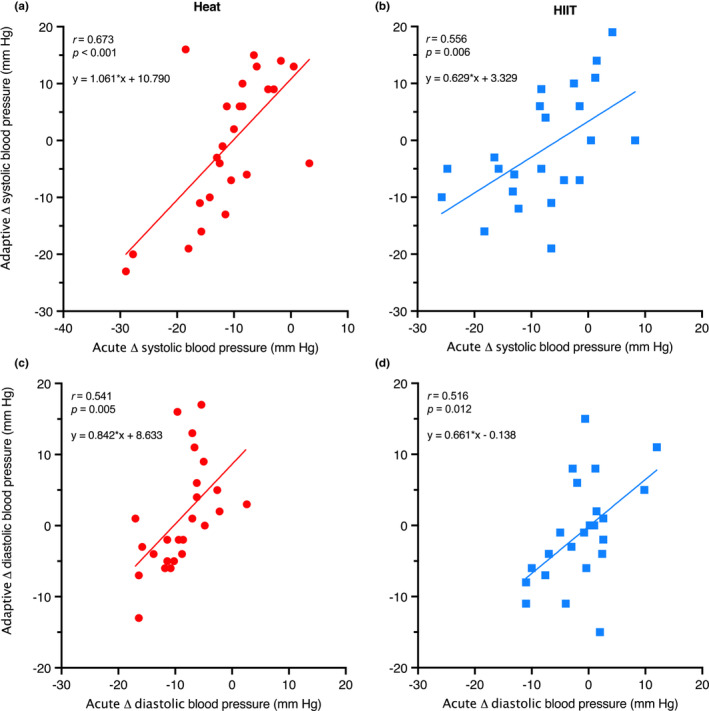
The magnitude of change (Δ) between pre‐session and average post‐session systolic (a & b – top graphs) and diastolic BP (c & d – bottom graphs) (acute; *x*‐axis), and the magnitude of change (Δ) in resting systolic BP and diastolic BP across the intervention (adaptive; *y*‐axis), for Heat (red) and HIIT (blue) participants.

The BP response to an acute exposure of hot‐water immersion or HIIT in the first intervention session was moderately correlated with the adaptive response (Tables 3 and 4, Figures 10 and 11, respectively). Specifically, for Heat, the time‐averaged and matched change in systolic and diastolic BP during recovery had the highest correlation with the adaptive change in resting systolic BP (*r* = 0.673, *p* < 0.001; 95% CI 0.38, 0.84) and diastolic BP (*r* = 0.541, *p* = 0.005; [0.19, 0.77]). For HIIT, the 5‐min recovery matched BP had the highest correlation with adaptive BP change, for both systolic BP (*r* = 0.618, *p* = 0.001; [0.29, 0.82]) and diastolic BP (*r* = 0.630, *p* = 0.001; [0.30, 0.82]).

**TABLE 3:** Correlation between blood pressure response immediately following acute hot‐water immersion in week one and adaptive resting blood pressure response across the intervention in Heat participants.

Variable	5 min	10 min	20 min	Average
Time point during recovery				
Systolic blood pressure	0.531	0.561*	0.596*	0.673*
Diastolic blood pressure	0.277	0.402*	0.275	0.541*

*Note*: Data are the Pearson correlation coefficient between adaptive blood pressure change (i.e., pre‐intervention – post‐intervention) and blood pressure measurement at each time point, or between adaptive change and average change in blood pressure measurement during recovery.

* *p* < 0.05.

**TABLE 4:** Correlation between blood pressure response immediately following high‐intensity interval training in week one and adaptive blood pressure response across the intervention in HIIT participants.

Variable	5 min	10 min	20 min	Average
Time point during recovery				
Systolic blood pressure	0.618*	0.381	0.320	0.556*
Diastolic blood pressure	0.630	0.393	0.539	0.516*

*Note*: Data are the Pearson correlation coefficient between adaptive blood pressure change (i.e., pre‐intervention – post‐intervention) and blood pressure measurement at each time point, or between adaptive change and average change in blood pressure measurement during recovery.

* *p* < 0.05.

#### 3.3.4 Glycemic control

**FIGURE 12:**
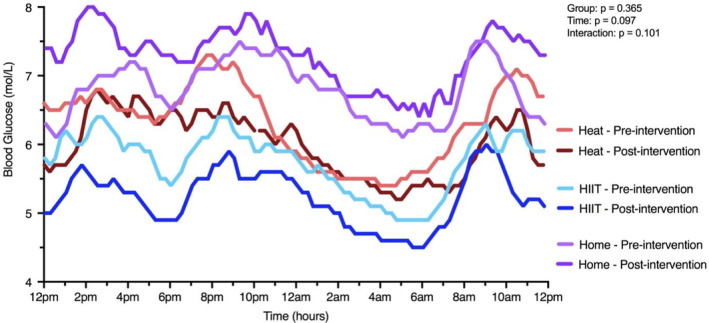
Mean 24‐h blood glucose pre‐ and post‐intervention for a sub‐set of Heat (*n* = 10), HIIT (*n* = 8), and Home (*n* = 7) participants.

HbA1c was unchanged in either group across the intervention (Table 5 and Figure 12). In a sub‐analysis of participants with HbA1c >40 mmol mol^−1^, a main effect of time was observed (*p* = 0.038). For CGM analysis, area under the curve trended lower across the intervention (interaction effect: *p* = 0.077), but no reliable changes were observed.

**TABLE 5:** Glycemic control in Heat, HIIT, and Home groups.

Variable	Heat		HIIT		Home		Statistical significance		
	PRE	POST	PRE	POST	PRE	POST	Group	Time	Interaction
Venous blood sample									
HbA1c (mmol mol^−1^)^a^	42 (13)	41 (10)	39 (10)	39 (8)	39 (11)	39 (12)	0.663	0.901	0.310
HbA1c >40 (mmol mol^−1^)^a^	54 (16)	50 (11)	48 (11)	46 (9)	54 (20)	54 (22)	0.648	0.038	0.328
Continuous blood glucose monitoring									
Daily average (mmol L^−1^)^a^	6.3 (1.5)	6.0 (1.2)	5.8 (2.2)	5.2 (1.1)	6.8 (4.5)	7.3 (5.9)	0.365	0.097	0.101
Night average (mmol L^−1^)^a^	5.8 (1.4)	5.6 (1.2)	5.4 (2.1)	5.0 (0.9)	6.7 (4.5)	7.0 (5.7)	0.313	0.183	0.449
TIR (min)^a^	1236 (236)	1254 (253)	1091 (270)	1205 (228)	1093 (549)	1106 (500)	0.547	0.246	0.595
CV (%)^a^	21.1 (7.8)	21.1 (12.2)	19.5 (3.3)	18.8 (3.3)	15.5 (1.5)	14.9 (2.5)	0.161	0.222	0.975
GMI (mmol mol^−1^)^a^	42 (7)	41 (6)	40 (10)	37 (5)	45 (21)	47 (28)	0.653	0.338	0.437
AUC^a^	599 (141)	568 (118)	546 (207)	496 (103)	647 (427)	691 (561)	0.366	0.120	0.077

*Note*: Variables are presented as mean (SD) and analyzed with a mixed model ANOVA.

Abbreviations: AUC, area under the curve; CV, coefficient of variation; GMI, glucose management indicator; POST, post‐intervention; PRE, pre‐intervention; SD, standard deviation; TIR, time in range.

^a^ Log transformed for statistical analysis. Night was defined as 10 p.m. – 6 a.m.

## 4 DISCUSSION

The main findings of this study were (1) A single exposure of hot‐water immersion elicited a larger acute hypotensive response than HIIT (mean arterial BP reduction from baseline: −8 mm Hg vs. −4 mm Hg), and importantly, this *acute* effect persisted across 12 weeks of repeated exposure; (2) After 12 weeks, both heat therapy and HIIT elicited potent adaptive hypotensive effects on resting BP (≥7 mm Hg reduction in systolic BP) and reductions were larger in participants who had hypertension; (3) A relationship between the acute and adaptive hypotensive response was observed for both interventions, with a larger acute post‐exposure hypotensive effect moderately associated with a larger decrease in resting BP across the intervention; and (4) lastly, neither intervention improved glycemic control.

### 4.1 How do acute cardiovascular responses differ between Heat and HIIT in patients with severe lower‐limb osteoarthritis?

#### 4.1.1 Blood pressure

Both hot‐water immersion and a bout of HIIT had potent anti‐hypertensive effects, with large acute reductions in systolic (10–12 mm Hg), diastolic (4–7 mm Hg) and mean arterial (4–8 mm Hg) BP. The reductions in diastolic and mean arterial BP, but not systolic BP, were larger following hot‐water immersion compared with an acute bout of HIIT.

During hot‐water immersion, systolic BP dropped within 10 min of exposure and remained below baseline for the remainder of the immersion and recovery period. Similarly, substantial reductions have been reported before: Thomas et al. (Thomas et al., 2017) showed that 30 min of lower‐limb hot‐water immersion had a potent hypotensive effect during exposure in healthy participants (systolic BP: 144 to 104 mm Hg; diastolic BP: 71 to 57 mm Hg) and patients with peripheral arterial disease (158 to 121 mm Hg; 77 to 64 mm Hg); the effect was still evident 3 h post‐immersion for both healthy controls (vs. baseline: −14 & ‐4 mm Hg) and patients with peripheral arterial disease (vs. baseline: −5 & ‐6 mm Hg). A recent study in young, healthy individuals examined the hypotensive response following 60 min of thermal‐load matched hot‐water immersion or aerobic exercise (Francisco et al., 2021). The drop in systolic BP was smaller than in the current study, but larger for diastolic BP with both hot‐water immersion (systolic BP: −5 mm Hg; diastolic BP: −9 mm Hg) and exercise (−5 mm Hg; −9 mm Hg) up to 20 min after exposure.

This is the first study to report the magnitude of the acute hypotensive response during hot‐water immersion is not different for the same exposure duration across 12 weeks of repetition. While we did not measure the duration post‐exposure for which this effect persisted, significant reductions up to 3‐h post‐exposure have been reported (Thomas et al., 2017). The occurrence of a prolonged post‐immersion hypotensive period has important health implications in that if repeated regularly, cardiovascular and neurological risk is potentially significantly reduced for several hours during that day. Even a 5 mm Hg pharmacological reduction in resting BP reduces major cardiovascular event by ~10% over a ~4 year follow‐up period (Rahimi & Treatment, 2021). So, for patients with hypertension, decreases in BP for a proportion of the day are likely to be beneficial (Brito et al., 2018; Wallace et al., 1999).

Upper‐limb HIIT also induced a post‐exercise hypotensive response, persisting for the 20 min period over which it was measured. HIIT has previously been shown to have a greater hypotensive effect than isocaloric continuous moderate‐intensity exercise in adults with hypertension (Pimenta et al., 2019). Pimenta et al. (2019), showed at 60 min post‐exercise, systolic BP was lower following HIIT (−11 ± 12 mm Hg), compared with continuous moderate‐intensity exercise training (CMIET; −7 ± 10 mm Hg; *p* ≤ 0.001); diastolic BP decreased with HIIT (−7 ± 8 mm Hg), but not significantly with CMIET (−4 ± 8 mm Hg; *p* = 0.141). Systolic BP and diastolic BP may be reduced for up to 11 and 4 h respectively following an acute CMIET bout in adults with hypertension (Wallace et al., 1999), so it is likely the effect derived from HIIT also extends beyond commonly measured recovery periods (20–60 min). The results of the current study also support the use of upper limb exercise for eliciting post‐exercise hypotension.

Following exercise, baroreceptor resetting, reduced sympathetic nerve activity, reduced vasoconstriction and active vasodilation all contribute to early and sustained post‐exercise hypotension. Depending on the type and intensity of exercise, post‐exercise hyperemia can be present for up to 20 min, with increased shear stress and other vasodilatory signals from exercise carrying over into early recovery (Halliwill et al., 2013; Tinken et al., 2010), contributing to the initial post‐exercise hypotension. Sustained post‐exercise hypotension appears to be mediated by activation of histamine H_1_ and H_2_ receptors, with blocking of these receptors reducing post‐exercise vasodilation by ~80% and post‐exercise hypotension by ~65% (Halliwill et al., 2013; McCord & Halliwill, 2006). Hypotension also triggers a cascade of hormonal responses to promote sodium and water retention, resulting in a supercompensation of plasma volume (Convertino et al., 1980; Hayes et al., 2000) and also potentially angiogenesis (Halliwill et al., 2013). Plasma volume expansion and angiogenesis are two of the key training adaptions associated with exercise for improving cardiorespiratory fitness (Hopper et al., 1985), both of which may contribute to long‐term BP reductions. Mechanisms contributing to the adaptive anti‐hypertensive effects with heat therapy are likely similar to exercise and may include upregulation of heat shock proteins, endothelial nitric oxide synthase and reduced inflammatory and oxidative stress response (Ely, Francisco, et al., 2019; Hesketh et al., 2019; McClung et al., 2008; Pritchard Jr. et al., 2001).

#### 4.1.2 Heart rate

Heat stress triggers a stress response that elevates heart rate via a downregulation of parasympathetic activity (Crandall et al., 2000; Crandall & Wilson, 2015) and concurrent increase in catecholamine activity and noradrenergic signaling (Rowell, 1990). Despite a 0.9°C increase in body core temperature, heart rate on average rose only ~16 b·min^−1^ above baseline. The modest increase in heart rate in the present study was similar to findings by Lucas et al. (2012) where older adults demonstrated a blunted response to supine heat stress. This contrasts with data from Francisco et al. in which a 20‐ and 60‐min immersion induced a ~35 and ~40 b·min^−1^ increase in heart rate, respectively (Francisco et al., 2021). Even waist‐deep water immersion (~42°C) elevated heart rate by 43 b·min^−1^ and 37 b·min^−1^ following 30‐min immersion in healthy controls and patients with peripheral arterial disease (Thomas et al., 2017). Impaired autonomic control and prescribed medications in the present study may partly explain discrepancies between studies.

HIIT – as intended – resulted in significant increases in exercising heart rate. In the final week of the intervention, participants were averaging ~90% heart rate reserve on completion of the final interval. The relevance of this is that despite the presence of severe lower‐limb osteoarthritis, participants were still able to perform physiologically stressful exercise. Besides cardiovascular risk reduction, regular exercise is essential for maintaining physical and mental health and optimizing perioperative risk for when patients inevitably undergo surgery (Roxburgh et al., 2023; Taylor et al., 2004). Health and exercise professionals should use these findings to educate their patients that exercise, specifically HIIT, utilizing upper limbs is feasible and effective for achieving these goals.

Rate pressure product, as an indicator of myocardial oxygen demand, (the product of systolic BP and heart rate), was more than double with exercise compared with hot‐water immersion. So despite similar hemodynamic changes (e.g., increased cardiac output, post‐exposure hypotension) (Francisco et al., 2021), the oxygen demands placed on the heart are distinctly lower with heat therapy. Particularly for those with high cardiovascular risk, the ability to achieve significant physiological stress (i.e., increased body core temperature, favorable shear profiles and post‐exposure hypotension) without high cardiovascular strain is advantageous.

### 4.2 What were the adaptive effects of heat therapy and HIIT on cardiometabolic health?

Both passive heat therapy and HIIT incorporating the upper limbs had potent effects on BP. Specifically, across the intervention, resting BP decreased 9 mm Hg (systolic) and 4 mm Hg (diastolic) with passive heat therapy, and 7 mm Hg and 3 mm Hg with HIIT. A recent meta‐analysis of 15 intervention studies on passive heat therapy showed a modest but consistent reduction in mean arterial BP, systolic BP, and diastolic BP of −6, −4 and −4 mm Hg, respectively in normotensive participants (Pizzey et al., 2021). Similarly, with exercise training (Cornelissen & Fagard, 2005; Naci et al., 2019), participants with elevated BP have greater capacity for therapeutic benefit. Akerman et al. (2019) reported a 7 mm Hg reduction in systolic BP following 12 weeks of hot‐water immersion in patients with peripheral arterial disease; 90% of these participants had hypertension, potentially contributing to the larger decrease in systolic BP. Masuda and others (Masuda et al., 2004) also reported a larger reduction (12 mm Hg) in systolic BP in participants with at least one cardiovascular disease risk factor after 14 sessions of heat (Waon) therapy.

To our knowledge, this is the first study to have investigated the effect of an upper‐limb HIIT intervention on resting BP. We showed that 7 mm Hg and 3 mm Hg reductions in systolic BP and diastolic BP could be achieved through regular upper‐limb HIIT. In fact, the magnitude of the reductions is larger than previously reported. A meta‐analysis of 105 aerobic exercise training interventions reported a decrease in systolic BP of 3.5 mm Hg and diastolic BP of 2.5 mm Hg (Cornelissen & Smart, 2013). More recently, Naci and colleagues (Naci et al., 2019) compared the effectiveness of 391 exercise interventions and anti‐hypertensive pharmaceuticals on systolic BP. The authors revealed that exercise training reduced systolic BP by ~5 mm Hg [−6, −4], compared to an ~9 mm Hg reduction (−9.6, 8.0) with medication. The adaptive reduction of systolic BP observed in this current study was similar to randomized controlled trials (≥4 weeks duration) investigating the effect of anti‐hypertensive pharmaceuticals. Specifically, ace inhibitors (−7 mm Hg [−9, −6]), calcium channel blockers (−11 mm Hg [−12, −9]), and diuretics (−8 mm Hg [−10, −7]) have similar effects on systolic BP as heat therapy or HIIT in the present study (Naci et al., 2019). Neither intervention induced a decrease in resting heart rate.

Glycemic control was not significantly affected by either intervention. These findings are likely due to inadequate power for this comparison and the heterogeneity of participants; a large number possessed a “normal” HbA1c (i.e., <39 mmol mol^−1^), and changes in HbA1c are less pronounced in non‐diabetics compared to patients with pre‐diabetes or diabetes (Cavero‐Redondo et al., 2018). We conducted an exploratory post hoc analysis in participants who met American Diabetes Association criterion for pre‐diabetes (i.e., HbA1c >39 mmol mol^−1^) (American Diabetes Association, 2014). In this sub‐sample, there was a main effect of time, but despite a 4 mmol mol^−1^ reduction in the Heat group (*n* = 9), there was no statistically significant interaction. In a well‐cited letter to the editor, Hooper (Hooper, 1999) reported reduced HbA1c from 100 to 89 mmol mol^−1^ in eight patients with type 2 diabetes with 3 weeks of hot‐water immersion, however, there was no control group.

Another possibility is that passive heat therapy is not effective for improving glycemic control, or may even be deleterious. Acute passive heat exposure results in acute glucose intolerance in non‐diabetic and diabetic individuals (Behzadi et al., 1985; Leicht et al., 2019; Maley et al., 2019), with peripheral tissue glucose uptake reduced in response to hot‐water immersion (but not thermoneutral exposure) (Maley et al., 2023). It is plausible the sympathetic response that occurs during hot‐water immersion increases hepatic glucose output; however, the metabolic demands are not sufficient to utilize this additional substrate.

### 4.3 Do acute blood pressure responses predict adaptive changes to passive heat therapy or HIIT?

In the last decade, there has been interest in the predictive value of the response to an acute exposure to indicate likely adaptive responses; no previous study has investigated this relationship with hot‐water immersion or HIIT. Our analyses revealed that BP measured 5 min after an acute bout of HIIT had the highest correlation with adaptive BP changes across the intervention (*r* ≥ 0.62). Meanwhile with hot‐water immersion, an average of the 20‐min recovery BP was the best predictor (*r* ≥ 0.54). Liu and colleagues (Liu et al., 2012) reported that the greater the hypotensive response following a single exercise session, the greater the drop in systolic BP (*r* = 0.89, *p* < 0.01) and diastolic BP (*r* = 0.75, *p* < 0.01) over 8 weeks of repeated exercise. Heckstden et al. (Hecksteden et al., 2013) performed a similar study in healthy untrained middle‐aged adults (49 ± 7 years). Following 4 weeks of continuous moderate‐intensity exercise training (4 sessions per week; 45 min per session; 60% HRR on a treadmill), the adaptive change in systolic BP and diastolic BP was correlated with the magnitude of post‐exercise hypotension measured 1 h following the initial exercise session (*r* = 0.77, *p* = 0.003; *r* = 0.66, *p* = 0.020). While associations for systolic and diastolic BP in the current study were not as strong as those observed by Liu et al. (2012), they were similar to those of Hecksteden et al. (2013). Thus, monitoring responses to acute exposure likely has clinical utility for individualizing exercise or passive heat therapy prescription. For example, manipulation of exercise parameters (i.e., type, intensity, duration) to achieve a post‐exposure hypotensive response may increase the probability of longer‐term adaptive responses with repeated bouts. Alternatively, this approach may more rapidly identify individuals who do not respond to a particular therapy as a front‐line approach (i.e., no post‐exposure hypotensive response), allowing clinicians to pivot more rapidly to alternative treatments (e.g., pharmacological).

## 5 LIMITATIONS

The findings of this study should be considered in light of the following limitations. These data are from a large clinical trial that was primarily designed to detect changes in cardiorespiratory fitness. Although methodologically robust, the variables in this study were secondary outcome measures and should be interpreted in this light. Blood pressure measurements (although measured in duplicate or triplicate) were taken during a single exposure or measurement session; regression to the mean is possible as a result. Therefore, adequately designed studies are necessary to confirm the findings of this study.

## 6 CONCLUSIONS

Passive heat therapy and upper‐limb HIIT were both effective for lowering systolic and diastolic BP, acutely and chronically. Exercise is well‐known to have this effect and we have contributed evidence of the efficacy of a novel exercise mode. Additionally, heat therapy is emerging as a potent anti‐hypertensive therapy, and herein, we demonstrate significant reductions in BP with a minimally arduous protocol (~90 min per week). The magnitude of this effect in the current study was similar to a 12‐week course of many commonly prescribed anti‐hypertensive pharmaceuticals. Additionally, the magnitude of BP reduction following acute hot‐water immersion and upper‐limb HIIT was associated with reductions in resting BP with repeated exposure across 12 weeks. Heat therapy and HIIT utilizing the upper limbs may be appropriate treatments for individuals who are challenged by traditional exercise – such as osteoarthritis – or are resistant to pharmaceutical anti‐hypertensive treatment.

## AUTHOR CONTRIBUTIONS

Study conception and design: Brendon H. Roxburgh, David Gwynne‐Jones, James D. Cotter, and Kate N. Thomas. Data collection, analysis, and interpretation: Brendon H. Roxburgh, Holly A. Campbell, James D. Cotter, Michael J. A. Williams, and Kate N. Thomas. Drafting of original manuscript: Brendon H. Roxburgh and Kate N. Thomas. Critical revisions of the work for important intellectual content: Brendon H. Roxburgh, James D. Cotter, Holly A. Campbell, Ulla Reymann, David Gwynne‐Jones, Michael J. A. Williams, and Kate N. Thomas. Final approval: Brendon H. Roxburgh, James D. Cotter, Holly A. Campbell, Ulla Reymann, David Gwynne‐Jones, Michael J. A. Williams, and Kate N. Thomas.

## FUNDING INFORMATION

This study was funded by the Health Research Council of New Zealand (grant number: 18/636 (KT)) and a Health Research South Start‐Up Award, University of Otago (KT). BR was supported by a University of Otago Doctoral Scholarship.

## CONFLICT OF INTEREST STATEMENT

No conflicts of interest, financial or otherwise, are declared by the authors.

## Supporting information


Data S1.
