# Supplementary material for: Acute and adaptive cardiovascular and metabolic effects of passive heat therapy or high‐intensity interval training in patients with severe lower‐limb osteoarthritis
Source: Physiol Rep. 2023 Jun 10;11(11):e15699. doi: 10.14814/phy2.15699 (PMC10257080; doi:10.14814/phy2.15699)
Supplement: Supplementary file 1 — Data S1. [file PHY2-11-e15699-s001.docx]

Table 1: Blood pressure and heart rate responses during an acute bout of hot-water immersion and 20 min seated recovery in session one, and during a session in the final week of the intervention

| **Variable** | **Exposure period** | | |  |  | | **Recovery period** | | | |  |
| --- | --- | --- | --- | --- | --- | --- | --- | --- | --- | --- | --- |
|  | **Pre** | **10 min** | **20 min** | **30 min** |  | **5 min** | | **10 min** | **15 min** | **20 min** | |
| *Week one* |  |  |  |  |  |  | |  |  |  | |
| SBP (mm Hg) | 122 (15) | 111 (15) | 112 (15) | - |  | 112 (12) | | 113 (14) | 112 (13) | 110 (13) | |
| DBP (mm Hg) | 73 (7) | 63 (8) | 59 (7) | - |  | 65 (8) | | 67 (8) | 67 (7) | 66 (7) | |
| MAP (mm Hg) | 89 (8) | 79 (8) | 77 (8) | - |  | 80 (8) | | 82 (8) | 82 (8) | 81 (8) | |
| Heart rate (b^.^min^-1^) | 77 (13) | 87 (12) | 92 (12) | - |  | 82 (14) | | 80 (13) | 79 (13) | 77 (13) | |
| *Final week* |  |  |  |  |  |  | |  |  |  | |
| SBP (mm Hg) | 122 (10) | 109 (12) | 110 (13) | 106 (11) |  | - | | - | - | - | |
| DBP (mm Hg) | 72 (7) | 61 (9) | 57 (8) | 55 (7) |  | - | | - | - | - | |
| MAP (mm Hg) | 89 (6) | 77 (9) | 75 (8) | 72 (7) |  | - | | - | - | - | |
| Heart rate (b^.^min^-1^) | 77 (11) | 82 (11) | 90 (12) | 93 (13) |  | - | | - | - | - | |

Variables are presented as mean (SD). DBP = diastolic blood pressure; MAP = mean arterial pressure; SBP = systolic blood pressure; PRE = pre-exposure. *n*=26.

Table 2: Blood pressure and heart rate responses during an acute bout of high-intensity interval training and 20 min seated recovery in session one, and during a session in the final week of the intervention

| **Variable** | **Exposure period** | | | | **Recovery period** | | | |  |
| --- | --- | --- | --- | --- | --- | --- | --- | --- | --- |
|  | **Pre** | **Peak-ex** |  | **5 min** | | **10 min** | **15 min** | **20 min** | |
| *Week one* |  |  |  |  | |  |  |  | |
| SBP (mm Hg) | 123 (13) | 165 (18) |  | 119 (14) | | 115 (13) | 114 (13) | 113 (13) | |
| DBP (mm Hg) | 76 (10) | 74 (12) |  | 72 (8) | | 73 (9) | 73 (8) | 72 (8) | |
| MAP (mm Hg) | 91 (10) | 104 (12) |  | 88 (8) | | 87 (8) | 87 (8) | 85 (9) | |
| Heart rate (b^.^min^-1^) | 74 (12) | 124 (23) |  | 88 (15) | | 87 (13) | 86 (13) | 83 (12) | |
| *Final week* |  |  |  |  | |  |  |  | |
| SBP (mm Hg) | 121 (9) | - |  | - | | - | - | - | |
| DBP (mm Hg) | 73 (6) | - |  | - | | - | - | - | |
| MAP (mm Hg) | 89 (6) | - |  | - | | - | - | - | |
| Heart rate (b^.^min^-1^) | 72 (12) | 125 (38) |  | - | | - | - | - | |

Variables are presented as mean (SD). DBP = diastolic blood pressure; MAP = mean arterial pressure; SBP = systolic blood pressure; Peak-ex = peak-exercise (i.e., maximum value during final interval); Pre = pre-exposure. *n*=25.
